# Five decades of clinical ABO-incompatible liver transplantation research: a bibliometric analysis

**DOI:** 10.1097/JS9.0000000000001987

**Published:** 2024-08-02

**Authors:** Maiwulanjiang Maimaiti, Alimujiang Mamuti, Yilizhati Azhati, Aliya Tulading, Yun-Fei Zhang, Jing Wu, Abudusalamu Tuersunmaimaiti, Chun-Hui Lv, Gang Yao, Amina Aierken, Tao Li, Tuerganaili Aji, Ying-Mei Shao, Hao Wen, Tuerhongjiang Tuxun

**Affiliations:** a Department of Liver and Laparoscopic Surgery, Center of Digestive and Vascular Surgery, 1st Affiliated Hospital of Xinjiang Medical University, Xinjiang Uyghur Autonomous Region; b Center of Organ Transplantation, 1st Affiliated Hospital of Xinjiang Medical University, Xinjiang Uyghur Autonomous Region; c Health Management Institute, Xinjiang Medical University, Xinjiang Uyghur Autonomous Region; d State Key Laboratory of Pathogenesis, Prevention, Treatment of High Incidence Diseases in Central Asia, Xinjiang Medical University, Urumqi, People’s Republic of China

Highlights

- This study is the first bibliometric analysis of ABO-incompatible liver transplantation.
- An increasing number of articles on ABO-incompatible liver transplantation have been published through past five decades.
- Japan is the most productive country in articles, researchers. In many Asian countries such as South Korea and Japan, ABO-incompatible liver transplantation has developed rapidly.
- Since the first case of ABO-incompatible liver transplantation was reported in 1979, most studies have focused on strategies to suppress the graft antibody-mediated immune rejection.

To gain a comprehensive understanding of the evolutions and hotspots, a bibliometric study was conducted using VOSviewer and CiteSpace software, focusing on articles related to ABOi-LT. We gathered literature from the Web of Science Core Collection, using keywords such as ‘ABO-incompatible’ or ‘ABO mismatch’ in conjunction with ‘liver transplant’. According to our included criteria (Fig. 1A), a total of 230 studies were finally included for further analysis. Notably, the number of publications has gradually increased since 1991, exhibiting an upward trend. The number of articles published with an average number of 4.6 annually, we indicate that ABOi-LT research is becoming an important topic in recent years.

**Figure 1:**
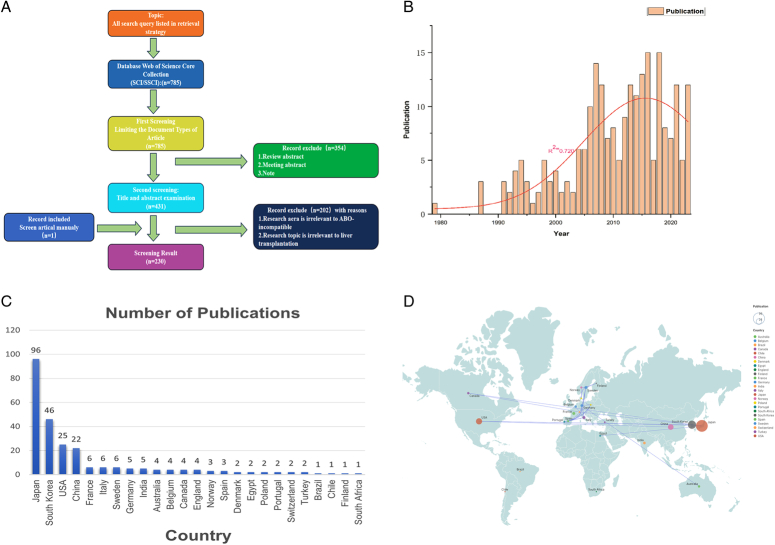
(A) The flowchart illustrates the study design and analysis approach used in the present study. (B) The chart shows the number of publications from 1973 to 2023. (C,D) Geographical distributions and network analysis of the 200 articles from 1973 to 2023.

More than 1000 scholars from 25 countries have contributed to the research of ABOi-LT. The Japan is at the forefront with 96 publications followed by South Korea (*n*=46, 20.09%) and the United States of America (*n*=26, 11.30%) (Fig. 1C). A total of 171 institutions have contributed to these articles and the top three institutions among of them were Kyoto University, University of Ulsan, and Keio University, respectively (Table 1). The three institutions contributed 65 articles in total, accounting for 28.38% of all articles and the No.1 and No.3 institutions are both from Japan, the prominent position of the Japan among all institutions underscores its powerful influence in this field.

**Table 1:** Distribution of top 10 institutions.

Rank	Institutions	No. of publications	Percent	Nation	Citations
1	Kyoto University	38	16.52%	Japan	1739
2	University of Ulsan	15	6.52%	Korea	410
3	Keio University	12	5.22%	Japan	383
4	Tohoku University	9	3.91%	Japan	265
5	Sungkyunkwan University	8	3.48%	Korea	123
6	Chang Gung University	8	3.48%	Taiwan, China	92
7	Kyushu University	7	3.04%	Japan	291
8	National Cancer Center	7	3.04%	Japan	94
9	Osaka University	6	2.61%	Korea	215
10	Kumamoto University	6	2.61%	Japan	98

The researchers who contribute to the field most are revealed in our study, we found that Uemoto Shinji (*n*=21) who rank the first with the most publications followed Hiroto Egawa (*n*=18) and Koichi Tanaka (*n*=15). The top 10 active authors are all from Asia, including seven from Japanese and three from South Korean (Table 2). Such a result indicates that Asian researchers have played a critical role and made significant contributions in the field of ABO-i LT.

**Table 2:** Distribution of top 10 authors and co-cited authors.

Rank	Author	Country	No. of publications	Co-cited author	Citation
1	Uemoto Shinji	Japan	21	Egawa, H	258
2	Hiroto Egawa	Japan	18	Demetris, AJ	112
3	Koichi Tanaka	Japan	15	Tanabe, M	101
4	Hwang Shin	Korea	12	Song, GW	90
5	Inomata Yukihiro	Japan	11	Gugenheim, J	76
6	Lee Sung-Gyu	Korea	8	Ikegami, T	67
7	Song Gi-Won	Korea	8	Haga, H	53
8	Kasahara Mureo	Japan	8	Farges, O	52
9	Haga Hironori	Japan	7	S-H, Kim	52
10	Oike Fumitaka	Japan	6	Hanto, DW	51

There are 78 academic journals published articles which are related to ABOi-LT, the Figure 2B showed that the number of publications of these journals ‘Transplantation Proceeding’ ranked the first (*n*=48), followed by ‘Transplantation’. Keywords are essential in an article as they distill and mirror the study’s main content. The keywords co-occurrence analysis can pinpoint frequently used keywords across various studies, can help researchers quickly identifying the focal points of research. Our result showed that except our index words the most frequent keywords in our study were ‘rituximab’, ‘plasmapheresis’, ‘splenectomy’, and ‘infusion therapy’ (Fig. 2C). Additionally, we used CiteSpace to identify burst keywords, which reveals the high frequency keywords during them prevailing time. As shown in Figure 2D, the most recent burst keyword is ‘desensitization’ in recent years. And confirmed the desensitization therapies are important in ABO-i LT field. Moreover, for further understanding the desensitization strategies of ABO-i LT, we summarized the effects of plasmapheresis, rituximab, intravenous immunoglobulin, and local infusion therapy, which are high frequency in keywords on suppressing immune rejection in ABO-i LT are fully described.

**Figure 2:**
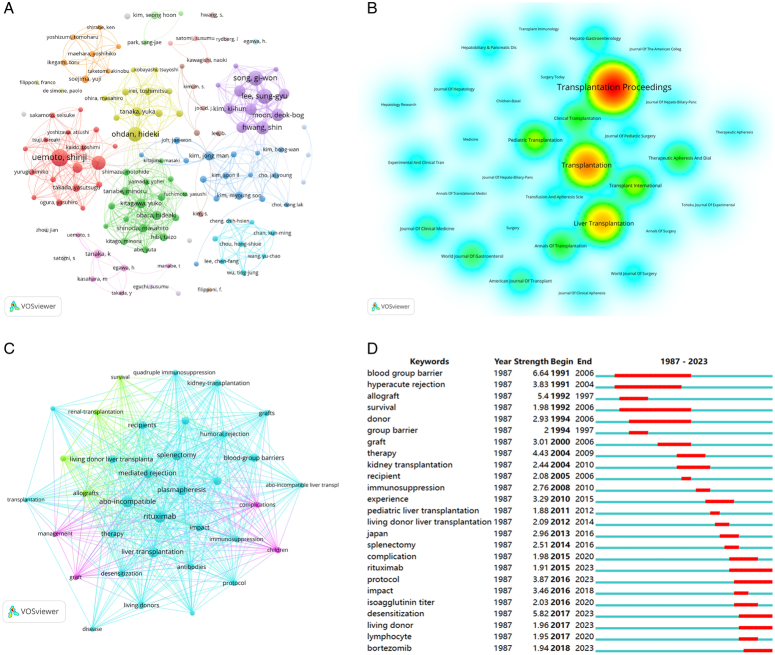
(A) A network map showing authors. (B) The journals of articles published from 1973 to 2023, and the size of circle represent the number of articles published, the more articles are published the circle’s color gradually approaches the red. (C) A network map of keywords. (D) Top 25 keywords with strongest citation burst.

At present, large number of studies have confirmed that the occurrence of antibody mediated rejection (AMR) after ABO-i LT is mainly related to lectin A/B and it has been reported that hepatic necrosis and intrahepatic biliary complications in ABOi-LT are closely related to high perioperative anti-A or anti-B antibody titers^1^. Therefore, plasmapheresis has been applied prior to LT in order to reduce antiblood antibodies to levels considered safe enough to improve the outcomes of ABOi-LT. It has been previously reported that the target of pretransplant antibody ABO-titer values following plasmapheresis were less than 1:8, 1:16, 1:32, or 1:64 to prevent posttransplant AMR^2^. However, the timing and frequency of plasma exchange initiation at major centers vary as per the experience of each transplant center and the individual patient.

Rituximab is a chimeric mouse/human anti-CD20 monoclonal antibody, which is mainly used in the treatment of lymphoma and some autoimmune diseases^3^. Its specific target is CD20 antigen on the surface of B cells, which is located on the surface of pre-B and mature B lymphocytes. Thus, rituximab is viewed as prophylactic therapy and the mechanism could be by reduction of precursor cells responsible for clonal expansion during AMR. Monteiro *et al*.^4^ reported the first use of rituximab in adult LT in 2003. Since then, several reports about using rituximab in ABO-i LT have been published, and drawn the conclusion that using rituximab is considered as an effective way to prevent the incidence rate of AMR after ABO-i LT. Meanwhile, most data show that a single dose of rituximab (375 mg/m^2^) is sufficient for suppressing B cells in the peripheral blood. Additionally, the large and multiple doses rituximab may add more the intensity of immunosuppression and neutropenia, resulting in an increased risk of cytomegalovirus infection^5^. Therefore, current studies show that most centers advocate a single administration of rituximab 375 mg/m^2^ in combination with other immunosuppression strategies 2 weeks before surgery.

Studies have proved that Intravenous Immunoglobulin (IVIG) has a wide range of immunomodulatory effects. Nowadays, IVIG also plays an inevitable role in graft protection by inhibiting Fc receptors on mononuclear phagocytes, directly neutralizing heterologous antibodies equivalent to blood type antibodies, inhibiting the expression of CD19 on activated B cells, inhibiting graft damage mediated by complement and allogeneic recognition T cells^6^. Therefore, some centers use IVIG as an effective mean to reduce anti-A or anti-B antibodies to make ABO-i LT successful and also in the treatment of AMR.

Local infusion therapy is the infusion of methylprednisolone, prostaglandin E1, and gab ethane mesilate into the transplanted liver through portal vein (or hepatic artery) to prevent intravascular thrombosis. introduced intraportal local infusion therapy in the early 2000s. Additionally, some research results showed that combined with local infusion therapy had increased the survival rate obviously^7^. While, some centers also put forward that the local infusion therapy not only increases the complexity of the operation, but also increases the occurrence of a series of postoperative catheter-related complications^8^. Therefore, local perfusion therapy has on effect on the survival or incidence of AMR in ABO-i LT is ambiguous.

At present, major transplant centers have gradually carried out ABO-i LT in pediatric. With the improvement and maturity of surgical strategies and desensitization programs, a large number of research data showed that the prognosis of ABO-i LT in pediatric patients is better than in adults. In 2009, Stewart *et al*.^9^ reported from the United Network for Organ Sharing (UNOS) registry analysis a superior graft survival rate for pediatric ABO-i LT compared to adult ABO-i LT. Mysore *et al*.^10^ who also analyzed the UNOS database came to the same conclusion and suggested that the anti-ABO antibody titer tailored approach. In their cohort children with anti-ABO antibody 1:32 were treated with plasmapheresis, rituximab, IVIG, and mycophenolate maintenance immunosuppression. While children with titer level 1:16 received only tacrolimus and corticosteroids. The results were excellent with 100% GS in the ABO-i LT group with a median follow-up of 3.3 years.

## Conclusion

To sum up, ABO-i LT can expand the pool of liver donors, ease the strain of lacking liver donors during emergency, and give patients who are waiting for LT newfound hope. To the best of our knowledge, this is the first bibliometric analysis that focused on the history and trends of ABO-i LT. Our results demonstrated that the desensitization is the main topics. Although many centers advocated that the strategy of rituximab combined with apheresis, IVIG, local infusion therapy have been used safely and widely used in ABO-i LT and the survival rate of ABO-i LT has also obtained satisfactory result, there are still some failure cases have been reported. Therefore, continuing to improve the current immunosuppression strategies or proposing new immunosuppression strategies to reduce the occurrence of AMR in ABO-i LT may be a key area for future research. And is an urgent need for researchers in the field.

## Ethical approval

Ethical approval was not need, as this was a pure bibliometric study.

## Consent

Not applicable.

## Source of funding

This work was supported by National Natural Science Foundation of China (No. 82270632, No. 82260411).

## Author contribution

M.M., A.M., A.T., Y.A., and T.T.: conceptualization; M.M., A.M., A.T., Y.F.-Z., A.T., Y.A., and C.H.-L.: data curation; M.M., A.M., A.T., J.W., and T.T.: formal analysis; M.M., A.M., Y.A., A.T., Y.F.-Z., and A.T.: investigation; M.M., A.M., A.T., and T.T.: methodology; T.T. and H.W.: supervision; M.M., A.M., Y.A., and A.T.: visualization; M.M., A.M., Y.A., and A.T.: writing – original draft; T.T., H.W., T.L., T.A., Y.M.-S., G.Y., and A.A.: writing – review and editing; T.T.: funding acquisition; T.T. and H.W.: project administration.

## Conflicts of interest disclosure

The authors declare no conflicts of interest.

## Research registration unique identifying number (UIN)

Not applicable.

## Guarantor

Tuerhongjiang Tuxun.

## Data availability statement

The data underlying this article are available in Web of Science Core Collection database. The data in this study are accessible in the public domain and not of a confidential nature. All the data could be contact with the corresponding author: turgunbay@163.com with scientific purpose.

## Provenance and peer review

Not commissioned, externally peer-reviewed.

## References

[1] Egawa H Oike F Buhler L. Impact of recipient age on outcome of ABO-incompatible living-donor liver transplantation. Transplantation 2004;77:403–411. 14966415 10.1097/01.TP.0000110295.88926.5C

[2] Yamamoto H Uchida K Kawabata S. Feasibility of monotherapy by rituximab without additional desensitization in ABO-incompatible living-donor liver transplantation. Transplantation 2018;102:97–104. 28938311 10.1097/TP.0000000000001956

[3] Piro LD White CA Grillo-López AJ. Extended Rituximab (anti-CD20 monoclonal antibody) therapy for relapsed or refractory low-grade or follicular non-Hodgkin’s lymphoma. Ann Oncol 1999;10:655–661. 10442187 10.1023/a:1008389119525

[4] Monteiro I McLoughlin LM Fisher A. Rituximab with plasmapheresis and splenectomy in abo-incompatible liver transplantation. Transplantation 2003;76:1648–1649. 14702545 10.1097/01.TP.0000082723.02477.87

[5] Song GW Lee SG Hwang S. ABO-incompatible adult living donor liver transplantation under the desensitization protocol with rituximab. Am J Transplant 2016;16:157–170. 26372830 10.1111/ajt.13444

[6] Urbani L Mazzoni A De Simone P. Treatment of antibody-mediated rejection with high-dose immunoglobulins in ABO-incompatible liver transplant recipient. Transpl Int 2007;20:467–470. 17263788 10.1111/j.1432-2277.2006.00447.x

[7] Egawa H Teramukai S Haga H. Impact of rituximab desensitization on blood-type-incompatible adult living donor liver transplantation: a Japanese multicenter study. Am J Transplant 2014;14:102–114. 24279828 10.1111/ajt.12520

[8] Oh J Kim JM. Immunologic strategies and outcomes in ABO-incompatible living donor liver transplantation. Clin Mol Hepatol 2020;26:1–6. 30909688 10.3350/cmh.2019.0023 PMC6940481

[9] Stewart ZA Locke JE Montgomery RA. ABO-incompatible deceased donor liver transplantation in the United States: a national registry analysis. Liver Transpl 2009;15:883–893. 19642117 10.1002/lt.21723

[10] Mysore KR Himes RW Rana A. ABO-incompatible deceased donor pediatric liver transplantation: Novel titer-based management protocol and outcomes. Pediatr Transplant 2018;22:e13263. 30070010 10.1111/petr.13263 PMC6197909

